# Severe Traumatic Brain Injury in Early Adulthood and Cerebral Amyloid Angiopathy: Still an Overlooked Association?

**DOI:** 10.1002/ccr3.71555

**Published:** 2025-12-22

**Authors:** Matija Zupan, Lara Straus, Matic Bošnjak, Tomaž Velnar, Senta Frol

**Affiliations:** ^1^ Department of Vascular Neurology University Medical Centre Ljubljana Ljubljana Slovenia; ^2^ Faculty of Medicine University of Ljubljana Ljubljana Slovenia; ^3^ Institute of Pathology University Medical Centre Ljubljana Ljubljana Slovenia; ^4^ Department of Neurosurgery University Medical Centre Ljubljana Ljubljana Slovenia

**Keywords:** case report, cerebral amyloid angiopathy, early childhood, neurology, traumatic brain injury, vascular

## Abstract

Recent research has increasingly recognized a potential link between severe traumatic brain injury (TBI) decades ago and the later development of cerebral amyloid angiopathy (CAA). Although the precise mechanisms linking these two pathologies are incompletely understood, there is a hypothesis that TBI may disrupt amyloid β (Aβ) turnover, with its resultant progressive accumulation within the walls of cerebral vessels. We present the case of a woman with biopsy‐confirmed CAA and a history of severe TBI in her early adulthood, who suffered three recurrent intracerebral hemorrhages (ICHs) in the right occipital region, during the course of 1 month. Two of the ICHs necessitated neurosurgical evacuation, and the patient showed a fairly good recovery. This case further extends our previously reported series on the relationship between preceding childhood TBI and the development of CAA. TBI increases amyloid precursor protein production enhancing Aβ levels and promoting chronic blood–brain barrier dysfunction, impairing Aβ clearance. The glymphatic system and intramural periarterial drainage pathways may be compromised following TBI. Additionally, the inflammatory response to TBI promotes vascular oxidative stress and endothelial dysfunction, which may further exacerbate Aβ accumulation. A repeated ICH may be associated with a much worse clinical outcome, necessitating prolonged meticulous observation after the first bout of an ICH in these patients. Further research is needed to clarify TBI's role in CAA progression.

## Key Clinical Message

Severe traumatic brain injury (TBI) as a risk factor for cerebral amyloid angiopathy development remains under‐recognized in clinical practice. In patients presenting with intracerebral hemorrhage (ICH) in atypical locations, especially when typical causes of spontaneous ICH are excluded, clinicians should carefully assess for a history of severe previous TBI.

## 1 Introduction

Cerebral amyloid angiopathy (CAA), primarily affecting older adults, is characterized by the deposition of β‐amyloid (Aβ) in the walls of small‐ and medium‐sized cerebral and leptomeningeal blood vessels, often resulting in recurrent lobar intracerebral hemorrhages (ICH) [1]. Clinically, CAA is associated with ICH, cognitive decline, dementia, and transient focal neurological deficits (TFNEs) [2]. Neuroimaging typically reveals cortical microbleeds, cortical superficial siderosis, and lobar ICH in atypical locations. PET imaging can detect diffuse cortical amyloid deposits, while cerebrospinal fluid (CSF) analysis may reveal reduced Aβ levels. However, a definitive diagnosis requires histopathological examination of brain tissue, usually obtained via biopsy or autopsy, confirming Aβ deposits in leptomeningeal and cortical vessels [2].

Emerging evidence points to a potential association between severe traumatic brain injury (TBI) in childhood and the subsequent development of CAA [3]. While the precise mechanisms underlying this relationship are not fully understood, it is hypothesized that TBI may disrupt Aβ metabolism, resulting in its progressive accumulation within cerebral vessels.

We present the case of a female patient with recurrent ICH and a history of severe TBI in early adulthood, in whom brain biopsy confirmed the diagnosis of CAA. This case complements our previously reported series of four patients [3], reinforcing the growing evidence of an association between TBI and CAA.

## 2 Case History and Examination

A 63‐year‐old right‐handed female presented to the neurological emergency department (NED), University Medical Centre Ljubljana, in January 2025 with acute headache and vomiting, followed by a rapid decline in consciousness (GCS 3). Her past medical history included a severe TBI at 21 years of age, after which her professional life was reportedly uneventful (premorbid modified Rankin score (mRS) of 0), without known chronic conditions. Upon arrival at the NED, she was analgosedated, intubated and mechanically ventilated. Neurological examination revealed a non‐reactive dilated right pupil, while corneal and vestibulo‐ocular reflexes remained intact. Her arterial blood pressure was 160/90 mmHg, and ECG showed sinus rhythm. Laboratory tests, including coagulation studies and tumor markers, were normal, and chest X‐ray findings were unremarkable.

## 3 Methods (Investigations and Treatment)

**FIGURE 1:**
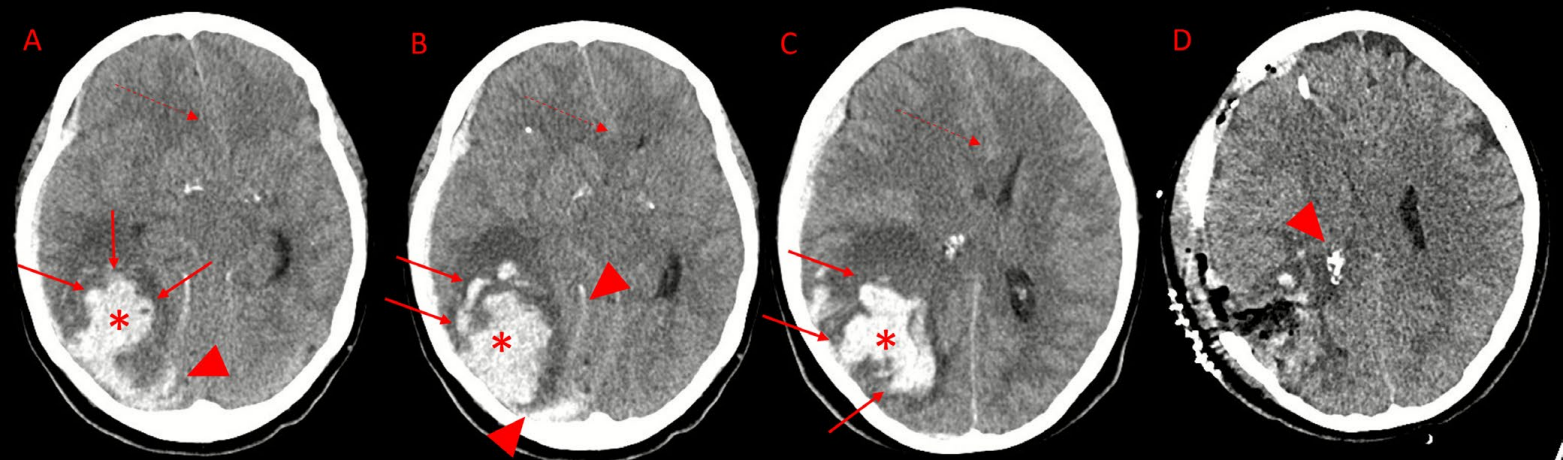
Preoperative and postoperative CT images. Unenhanced CT images show right occipital ICH (*) with fingerlike projections (arrows) and subarachnoid hemorrhage (SAH) (arrowheads) before surgery (A–C). Cerebral oedema and midline shift are evident. Post‐surgery CT (D) shows decompressive craniectomy, ICH evacuation, reduced oedema, and remnants of SAH (arrowhead).

**FIGURE 2:**
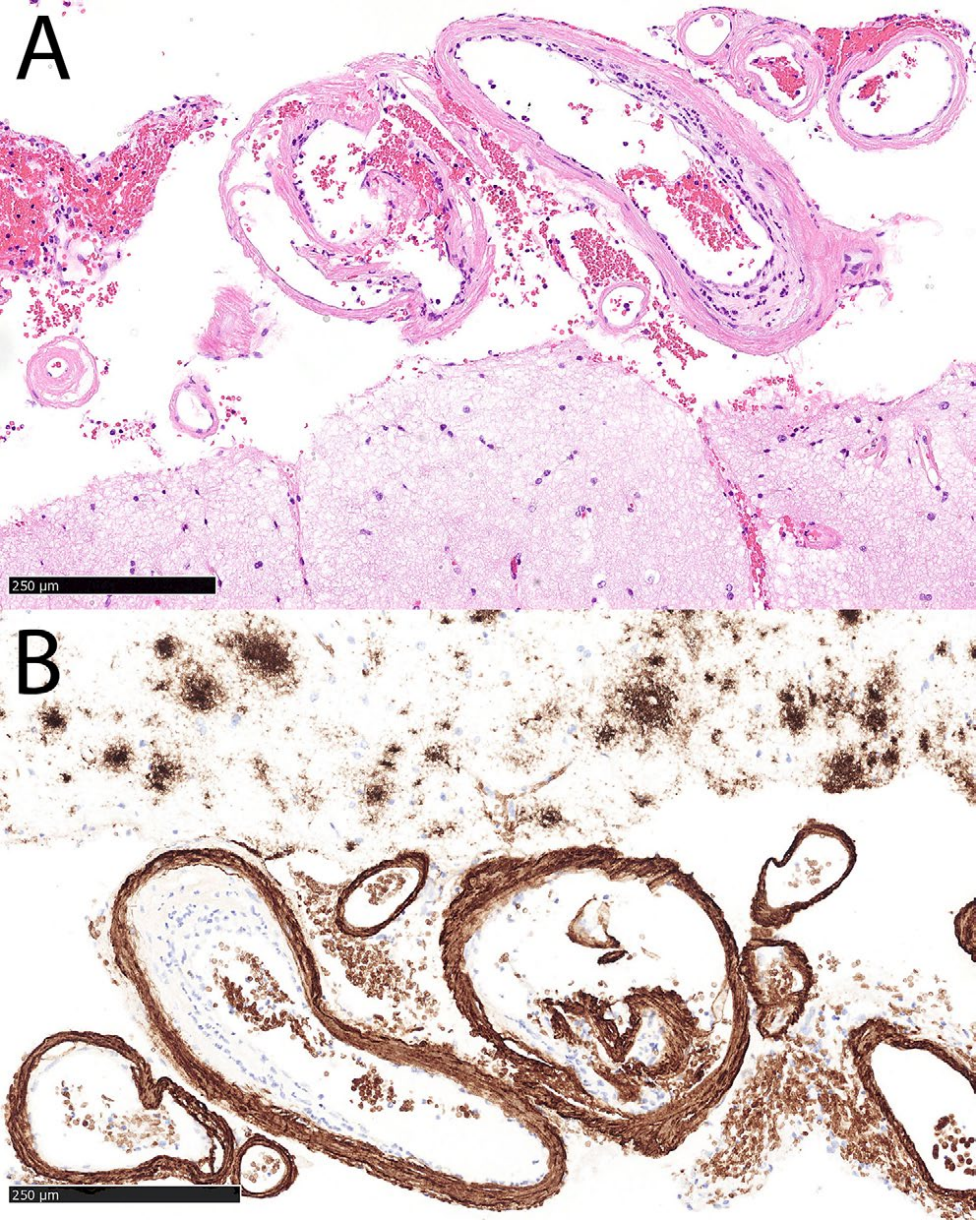
Histopathological findings. Hematoxylin & Eosin stain reveals lamellar eosinophilic deposits in the walls of leptomeningeal arteries and cortical arterioles (A). The deposits correspond to Aß by immunohistochemistry (anti‐Aß stain) (B). Note also diffuse neuritic plaques present throughout the neuropil (upper half of the panel) and the non‐specific staining of erythrocytes.

A head computed tomography (CT) scan revealed a 6 × 5 × 6 cm ICH in the right hemisphere, accompanied by a subdural hematoma encircling the right cerebral hemisphere and tentorium, as well as transtentorial herniation. CT angiography was unremarkable; cerebral venous sinuses and major veins were patent. ICH was surgically evacuated (Figure 1). A cortical biopsy identified β‐amyloid (Aβ) deposits in the blood vessel walls (Figure 2), confirming a definitive diagnosis of CAA.

## 4 Conclusions and Results (Outcome and Follow‐Up)

After the first surgery, the patient was successfully extubated, regained consciousness, and was transferred to a regular ward with slight left‐sided hemiparesis (National Stroke Scale score (NIHSS) 6) for rehabilitation. Over the next month, she experienced two additional in‐hospital ICHs, with the first necessitating surgical intervention. After 3 months of neurorehabilitation, the patient showed modest improvement, without residual motor deficit. NIHSS at discharge was 0, and mRS was 1. Further rehabilitation was continued on an outpatient basis.

## 5 Discussion

This case represents our fifth documented patient with CAA and a history of severe TBI, further reinforcing the potential association between these conditions. Including our previously reported cases [3], the total number of childhood TBI cases linked to CAA now exceeds the 13 cases described by Suzuki et al. [4], underscoring the need for further research into this relationship.

The pathophysiology linking severe TBI to CAA is not fully understood but likely involves a multifactorial process. TBI has been shown to disrupt Aβ metabolism by increasing amyloid precursor protein production and impairing Aβ clearance mechanisms by means of chronic blood–brain barrier (BBB) disruption with resultant accelerated Aβ deposition [4, 5]. The glymphatic system and intramural periarterial drainage pathways, crucial for Aβ clearance, may be compromised following TBI, facilitating Aβ deposition in vessel walls [4, 6]. Additionally, the inflammatory response to TBI with persistent activation of microglia and astrocytes promotes vascular oxidative stress, endothelial dysfunction, and pericyte loss, which may further exacerbate Aβ accumulation. To summarize, a two‐hit hypothesis for CAA‐related ICH post‐TBI states that the first hit may cause amyloid dysregulation, chronic vascular inflammation, and microvascular injury, whereas age and progressive CAA pathology constitute the second hit, leading to further Aβ deposition, enhancing vessel fragility and thus promoting ICH in midlife [7].

Our case demonstrates two repeated ICHs during the subacute phase of the first ICH, which is not uncommon even in younger patients with CAA [8]. Even though our patient fared well, a repeated ICH may in general be associated with a much worse clinical outcome, necessitating prolonged meticulous observation after the first bout of an ICH in these patients. From the pathophysiological standpoint, the early ICH recurrence may be associated with a handful of different mechanisms. These are perivascular inflammation, pronounced in CAA‐related inflammation (which was, however, not the case in our patient), a disruption of the blood–brain barrier due to inflammatory cytokine release from the first ICH, further promoting perivascular inflammation, and impaired autoregulation with regional hypoperfusion and compensatory hyperaemia in the vicinity [9, 10]. What is more, incomplete clot organization and local fibrinolysis in the haematoma might destabilize the vessel wall further, leading to rebleeding at the original ICH site [9, 10].

Expanding case documentation through international registries would be crucial for unraveling these mechanisms and raising awareness among clinicians. Further research into this entity is essential to refine diagnostic approaches, understand its clinical implications, and improve patient care.

Severe TBI as a risk factor for CAA development remains under‐recognized in clinical practice, though it's increasing documentation in the literature highlights its potential significance. In patients presenting with ICH in atypical locations, especially when typical causes of spontaneous ICH are excluded, clinicians should carefully assess for a history of severe childhood, adolescence or early adulthood TBI. Early identification of this association could enhance diagnostic accuracy and guide management strategies.

Despite these advances, the pathophysiological mechanisms linking severe childhood, adolescence, and early adulthood TBI to CAA remain incompletely understood. Further research and empirical evidence are needed to validate the hypothesis that TBI may disrupt Aβ metabolism by upregulating amyloid precursor protein production and impairing the mechanisms responsible for Aβ clearance.

## Author Contributions

**Matija Zupan:** conceptualization, data curation, formal analysis, funding acquisition, investigation, methodology, resources, supervision, writing – original draft, writing – review and editing. **Lara Straus:** conceptualization, formal analysis, investigation, methodology, writing – original draft, writing – review and editing. **Matic Bošnjak:** data curation, investigation, methodology, validation, writing – original draft, writing – review and editing. **Tomaž Velnar:** data curation, formal analysis, investigation, methodology, writing – original draft, writing – review and editing. **Senta Frol:** conceptualization, data curation, investigation, methodology, resources, supervision, validation, writing – original draft, writing – review and editing.

## Funding

The authors have nothing to report.

## Ethics Statement

The present research complies with the guidelines for human studies, and the research was conducted ethically in accordance with the World Medical Association Declaration of Helsinki.

## Consent

Informed consent was obtained from the patient's relative involved in the study. Written consent was obtained from the patient's relative for the publication of this case report and the accompanying figures, which are part of the patients' records archived by the hospital.

## Conflicts of Interest

The authors declare no conflicts of interest.

## Data Availability

The authors have nothing to report.
